# Characterisation of Gut Microbiota in Ossabaw and Göttingen Minipigs as Models of Obesity and Metabolic Syndrome

**DOI:** 10.1371/journal.pone.0056612

**Published:** 2013-02-20

**Authors:** Rebecca Pedersen, Hans-Christian Ingerslev, Michael Sturek, Mouhamad Alloosh, Susanna Cirera, Berit Ø. Christoffersen, Sophia G. Moesgaard, Niels Larsen, Mette Boye

**Affiliations:** 1 Department of Bacteriology Pathology and Parasitology, National Veterinary Institute, Technical University of Denmark, Frederiksberg C, Denmark; 2 Department of Cellular & Integrative Physiology, Indiana University School of Medicine, Indianapolis, Indiana, United States of America; 3 Department of Veterinary Clinical and Animal Sciences, Faculty of Health and Medical Sciences, University of Copenhagen, Frederiksberg C, Denmark; 4 Novo Nordisk A/S, Maaløv, Denmark; 5 Danish Genome Institute, Aarhus C, Denmark; Teagasc Food Research Centre, Ireland

## Abstract

**Background:**

Recent evidence suggests that the gut microbiota is an important contributing factor to obesity and obesity related metabolic disorders, known as the metabolic syndrome. The aim of this study was to characterise the intestinal microbiota in two pig models of obesity namely Göttingen minipigs and the Ossabaw minipigs.

**Methods and Findings:**

The cecal, ileal and colonic microbiota from lean and obese Osabaw and Göttingen minipigs were investigated by Illumina-based sequencing and by high throughput qPCR, targeting the 16S rRNA gene in different phylogenetic groups of bacteria. The weight gain through the study was significant in obese Göttingen and Ossabaw minipigs. The lean Göttingen minipigs’ cecal microbiota contained significantly higher abundance of Firmicutes (*P<*0.006), *Akkermensia* (*P<*0.01) and *Methanovibribacter* (*P<*0.01) than obese Göttingen minipigs. The obese Göttingen cecum had higher abundances of the phyla Spirochaetes (*P<*0.03), Tenericutes (*P<*0.004), Verrucomicrobia (*P<*0.005) and the genus *Bacteroides* (*P<*0.001) compared to lean minipigs. The relative proportion of *Clostridium cluster XIV* was 7.6-fold higher in cecal microbiota of obese Göttingen minipigs as compared to lean. Obese Ossabaw minipigs had a higher abundance of Firmicutes in terminal ileum and lower abundance of Bacteroidetes in colon than lean Ossabaw minipigs (*P<*0.01). Obese Ossabaws had significantly lower abundances of the genera *Prevotella* and *Lactobacillus* and higher abundance of *Clostridium* in their colon than the lean Ossabaws. Overall, the Göttingen and Ossabaw minipigs displayed different microbial communities in response to diet-induced obesity in the different sections of their intestine.

**Conclusion:**

Obesity-related changes in the composition of the gut microbiota were found in lean versus obese Göttingen and Ossabaw minipigs. In both pig models diet seems to be the defining factor that shapes the gut microbiota as observed by changes in different bacteria divisions between lean and obese minipigs.

## Introduction

Obesity in humans has reached epidemic proportions worldwide, which is mainly due to a combination of inactive lifestyle and increased energy intake [1]. Obesity is a condition characterised by accumulation of fat in adipose tissue and a state of metabolic imbalance. The morbid conditions related to obesity such as abdominal obesity, insulin resistance and glucose intolerance, hypertension and dyslipidemia are together called the metabolic syndrome (MetS) [2]. Obesity and its co-associated morbidities namely cardiovascular disease, type-2 diabetes (T2D), fatty liver disease and hypertension pose a great economic burden in affected countries. Recently, the gut microbiota has been implicated as one of the influencing factors that further promotes obesity and metabolic disorders in obese subjects [3], [4]. The gut microbiota is affected by dietary changes and is considered to be an environmental factor affecting the energy balance and whole body metabolism which may contribute to obesity and its metabolic disorders [3], [5], [6]. Recent studies have shown an altered gut microbiota characterised by reduced diversity in diet induced obese and diabetic mice as well as diabetic humans [6]–[10]. Other studies in mice have demonstrated changes in the composition of the gut microbiota independently of the obese state and in response to high-fat/high-energy diet, suggesting other mechanisms that may contribute to obesity and metabolic syndrome [11], [12]. In addition, Cani and colleagues [13]–[15] have provided evidence of a causal role of gut microbiota in the low grade inflammation in the gut of mice. The gut microbiota may lead to low grade inflammation induced by the lipopolysaccharides (LPS) present on the outer membrane of Gram-negative bacteria, causing activation of the innate immune response [16]. This low grade inflammation is connected to low, but constant levels of LPS in the circulation and to increased levels of adiposity and insulin resistance [13]. Together these findings suggest that high-fat diet (HFD) and obesity are associated with gut microbiota dysbiosis leading to alterations of gut barrier and resulting in an increased level of circulating LPS and a low grade inflammation (reviewed in [17]). However most of these studies are performed in mice that are genetically obese (ob/ob mice) and due to the many differences between humans and mice these findings may not be translated directly to humans [18]. Therefore, other animal models may provide better models for obesity-gut-microbiota related studies. Pigs are generally considered to be excellent biomedical models for nutritional studies because their gastrointestinal tract and physiology have similarities to humans, making the pig an excellent animal model in obesity related studies [18]. Pigs have already been used in a few obesity studies to investigate their gut microbiota [19], [20]. Specifically, minipigs have a potential of becoming excellent animal models for gut microbiota related studies, due to their smaller size than domestic pigs and their tendency to become obese when fed *ad libitum*. In this study we investigate the composition of the gut microbiota in relation to diet, obesity and MetS in two pig models, Göttingen and Ossabaw minipigs. Female Ossabaw minipigs when fed high-fat/cholesterol, high-caloric diet develop symptoms of MetS with abdominal adiposity, glucose intolerance, insulin resistance and cardiovascular disease (CVD) [21]–[23] and therefore Ossabaw minipigs are considered one of the relevant animal models for obesity and MetS [24]. Göttingen minipigs are also used in diabetes research and are proposed to be good animal models for studying obesity and metabolic disorders caused by obesity [25]–[27]. To our knowledge the gut microbiota of Ossabaw and Göttingen minipigs have not been characterised previously, either in lean or obese states. Therefore, the objective of this study was to investigate the effect of obesity and diet on the composition of the gut microbiota in the two pig models of obesity. In Ossabaw minipigs the dietary regimen was very different between lean and obese animals and any differences found between these two groups will be a combined effect of obesity and different diet. In the Göttingen minipigs the diet composition did not differ much between lean and obese minipigs only the amount of food was different being, restrictive for the lean group and *ad libitum* for the obese group, and consequently group differences will most likely primarily be due to differences in obesity. We characterised the gut microbiota of Ossabaw and Göttingen minipigs by Illumina-based sequencing and further confirmed our findings using a high-throughput quantitative real time PCR (qPCR) platform.

## Materials and Methods

The studies in Göttingen minipigs were designed and performed at the University of Copenhagen in Denmark. The Ossabaw minipig studies were designed and performed at Indiana University School of Medicine. The diet and design of the two studies are different, therefore in this study we characterised the gut microbiota in the two pig models separately.

### Göttingen Minipigs Study

Female Göttingen minipigs (*n = *14) that were ovariectomized (Ellegaard Göttingen Minipigs A/S, Dalmose, Denmark) were subsequently housed at two years of age in facilities provided by the University of Copenhagen (Taastrup, Denmark) until they were euthanized at approximately 41–47 months of age. Of the 14 minipigs, seven were allocated as the obese group. The obese minipigs had been given therapeutic peptides for pharmacological studies prior to the obesity experiments but were subjected to a suitable washout period before the start of the present experiments. The lean group (*n = *7) were fed restrictively with 150 g of minipig chow (Altromin 9023, Christian Petersen A/S, Gentofte, Denmark), twice a day consisting of 19% protein, 8% fat and 73% carbohydrates. The obese group (*n = *7) were fed *ad libitum* Altromin 9033 (minipig chow, Christian Petersen A/S, Gentofte, Denmark) consisting of 25% protein, 11% fat, 64% carbohydrates. At approximately 122 days before the end of the experiment the animals were weighed biweekly and the body-fat composition was measured by dual energy x-ray absorptiometry scanning (DXA-scanning) (Hologic Explorer, Santax Medico, Aarhus, Denmark) at the end of experiment and the absolute mass of fat tissue was obtained by using the scanner software package [28]. All the animals were euthanized by pentobarbital. Colon and cecum samples with content were collected after the animals were euthanized. The samples were immediately frozen in liquid nitrogen and were subsequently stored at −80°C until further analyses.

### Ossabaw Minipigs Study

The samples provided for this study were obtained from female Ossabaw minipigs that belong to Indiana University School of Medicine and Purdue University breeding colony (West Lafayette, IN, USA). The lean Ossabaw minipigs (*n = *4) received a daily diet (standard chow) with a total caloric content of 2200 kcal and the calories provided by macronutrients were: protein 18.4%, fat 10.5% and carbohydrates 71.0%. The obese group of minipigs (*n = *4) received a high-energy diet of 4500–6000 kcal daily provided by: 16.3% protein, 42.9% fat and 40.8% carbohydrates (of which 20% came from fructose). Both groups were housed individually at an age of six months, were fed the respective diets for a period of 8–10 months, and were euthanized at the end of feeding experiments at an age of 14–16 months. All the minipigs were euthanized by cardiectomy while under anaesthesia by a combination of intramuscular injections of tiletamine-zolazepam (5 mg kg^−1^), xylazine (2.2 mg kg^−1^) and isoflurane (5%). Samples from colon and terminal ileum both including content were collected after the animals were euthanized and were immediately frozen in liquid nitrogen and subsequently stored at −80°C until further analyses.

### DNA Extraction and Purification

The DNA was extracted from colon and cecum digesta of lean (*n = *7) and obese (*n = *7) Göttingen minipigs and from colon and terminal ileum digesta of Ossabaw lean (*n = *4) and obese (*n = *4) minipigs. Digesta from colon, terminal ileum and cecum (200 mg each) was used for DNA extraction by Maxwell® 16 Cell DNA Purification Kit (Promega Corporation, Madison, WI, USA) according to manufacturer’s instructions with an additional bead beating step in order to disrupt the cell wall of Gram-positive bacteria.

### PCR Conditions

The DNA samples were used for amplification of the V5 region of the 16S rRNA gene. The PCR reactions were performed in duplicates of 49 µl per reaction consisting of 5 µL 5 X GoldTaq buffer (Applied Biosystems, Branchburg, NJ, USA), 20 µM of each forward and reverse primers, 2 µL dNTP (10 mM), 4 µL MgCl_2_ (25 mM), 0.5 µL AmpliTaq Gold® DNA Polymerase (5 U/µL) (Applied Biosystems), 31.5 µL H_2_O and 2 µl DNA of 100 ng µl^−1^. The PCR cycling conditions were an initial denaturing step of 94°C for 6 min, followed by 30 cycles of 94°C for 45 s, 57°C for 45 s, 72°C for 45 s and a final extension step at 74°C for 10 min. The amplification was performed using the V5 universal primer set (in house design), forward primer 804F (5′-GGATTAGATACCCNGGTAGTC-3′) and reverse primer 926R (5′-CCGTCAATTCCTTTRAGTTT-3′) using a *T3* Thermocycler (Biometra GmbH, D-37079 Göttingen, Germany). The quantity and quality of the resulting PCR products were then assessed on an Agilent 2100 Bioanalyzer using the Agilent DNA 1000 kit (Agilent Technologies, Waldbronn, Germany). The amplified DNA was purified for primers and detergents using a Qiagen MinElute PCR purification kit (Qiagen GmbH, Hilden, Germany) according to the manufacturer’s instructions and then pooled together and finally 3.6 ng of DNA were submitted to the National High-Throughput DNA Sequencing Centre at Copenhagen University, Denmark, for sequencing on an Illumina HiSeq™ 2000 platform.

### Sequence Analysis

The obtained sequences were sorted and normalized by BION-meta software [29] with the taxonomic classification according to the Greengene Database [30]. The software package can be obtained from: https://www.dropbox.com/sh/fumscuqpanqaqvu/_4H-XBxHQ. In brief, the sequences were initially de-multiplexed according to the primer and barcode sequences. Subsequently they were cleaned at both ends by removal of bases of a quality less than 96%. Identical sequences were further clustered and aligned into consensus sequences with a setting of 99.8% base quality. Consensus sequences of at least 40 nucleotides in length were further mapped into a table according to the individual barcodes. Finally, the consensus sequences were taxonomically classified against the Greengene SSU database using a word length of 8 and a match minimum of 30%. The top one percent of the obtained hits from the Greengene database was then used for taxonomical classification of the consensus sequences. The number of reads for each barcode was further normalized making it possible to do statistical analysis directly between individuals in the experiment. The Shannon-Weaver index of diversity (*H′*) [31] was further calculated manually to estimate the diversity of the bacteria at genus level from the obtained normalized sequences. Prior to analysis, data were log transformed for normalizations and analysed using one-way ANOVA or two-way ANOVA when appropriate using GraphPad prism version 5.00 for Windows (GraphPad software, San Diego, CA USA). The Kruskal Wallis [32] or Bonferroni’s post hoc test [33] was further used to test for differences among groups. *P*-values <0.05 were considered significant. The abundances of bacteria in figures are represented as mean and error bars representing standard deviations.

### Microbial Identification by 48.48 Dynamic Array

High throughput qPCR was performed by a 48.48 Dynamic Array Integrated Fluidic Circuits (Fluidigm, CA, USA). This platform combines 48 primers with 48 samples to run 2304 simultaneous qPCR reactions. In this chip, individual primer sets target the 16S rRNA gene DNA of different bacterial phylogenetic groups (Domain, Phylum, Class, Family, Genus and Species level) Hermann-Bank *et al*. (2013, *in prep*). Each sample consisted of 20 µmol l^−1^ forward and reverse primers, 1× Assay loading reagent (Fluidigm, PN85000746), 1× low EDTA TE buffer (AppliChem GmBH, Darmstadt Germany) and master mix consisting of; 20 X DNA binding dye sample loading reagent (Fluidigm, PN 100-0388), 20 X EvaGreen® DNA binding dye (Biotium, Heyward CA, USA,) and 2×Taqman master mix (Applied Biosystems). The 16S rRNA gene DNA concentrations were equilibrated to a concentration of 50 ng µl^−1^. In each run a non-template control (NTC) was included to detect any contamination or non-specific amplification. A melting curve analysis was performed for quality check of amplification and the non-specific reactions were excluded. The obtained C_T_ values from the finally accepted values were subsequently exported to Microsoft Excel for further analysis. The relative proportion of bacteria representing each taxon was calculated based on the Livak method [34]. Hence, the relative quantifications of the PCR signal of the target 16S rRNA gene in the obese minipigs in each group was related to that of the lean minipigs which were considered to harbour the reference composition of the gut microbiota. Fold differences in the different bacterial groups were subsequently calculated by 2^−ΔΔCt^ [34]. All the statistical analysis were performed on GraphPad Prism version 5.00 for Windows (GraphPad software, San Diego, CA,USA). The statistical analysis were performed by Mann-Whitney U test [35] and significant differences were considered when *P<*0.05.

## Results

### Phenotype of the Göttingen and Ossabaw Minipigs

The lean Göttingen minipigs (*n = *7) weighed 50.3±1.6 kg when the animals were euthanized, while the obese Göttingen minipigs (*n = *7) weighed 92.6±5.2 kg (Figure S1). Approximately four weeks prior to euthanasia and at the time of DXA scanning, the body weight of the lean minipigs was 49±1.6 kg with a body-fat percent of 26% (±4) and the obese minipigs had an average body weight of 87±5.3 kg with a body-fat percent of 42% (±4). The difference between body-fat and body weight in lean and obese minipigs was significant (*P<*0.001). There was no significant difference in blood triglycerides, total cholesterol and fasting blood glucose between lean and obese Göttingen minipigs. However there was a significant difference in fasting blood insulin levels between lean and obese Göttingen minipigs (*P<*0.01) (Table 1).

**Table 1:** Blood parameters in lean and Obese Ossabaw and Göttingen minipigs.

	Ossabaw minipigs		Göttingen minipigs		
	Lean (*n* = 4)	Obese (*n* = 4)	Lean (*n* = 7)	Obese (*n* = 7)	*P-*value
**Body weight (kg)**	60.4 (±6.6)	98.3 (±2.9)	50.3 (±1.6)	92.6 (±5.2)	<0.001
**Triglycerides (mmol/l)**	0.3 (±0.03)	0.5 (±0.03)	0.3 (±0.04)	0.5 (±0.07)	0.07
**Total cholesterol (mmol/l)**	2.0 (±0.1)	12.9 (±2.0)	1.8 (±0.1)	2.0 (±0.1)	0.2
**Fasting blood glucose (mmol/l)**	4.2 (±0.1)	4.6 (±0.2)	3.8 (±0.1)	3.7 (±0.4)	0.9
**Fasting insulin (pmol/l)**	15 (±3.5)	34.7 (±7.5)	141.2 (±32.4)	39.2 (±9.9)	0.01

The lean Ossabaw minipigs (*n = *4) at six months of age weighed 43.3±6.6 kg and by the end of the experiment, at an age of 16 months they weighed 60.4±7.5 kg (Figure S2). At six months of age the obese Ossabaw minipigs (*n = *4) weighed 42.8±2.4 kg and at the end of experiments they weighed 98.3±2.9 kg (Figure S2). The obese Ossabaw minipigs displayed MetS with total blood cholesterol and triglycerides higher than the lean Ossabaw minipigs (Table 1). The fasting blood glucose levels showed a trend toward being higher in obese than in lean Ossabaw minipigs and a similar trend was observed in blood insulin levels in obese (34.7 pmol l^−1^) and in lean (15 pmol l^−1^) Ossabaw minipigs (Table 1).

### Sequence Analysis

Obtained sequence files were analysed using the Bion-meta software [29]. A total of 603,926,924 reads were obtained from the Illumina sequencing. After de-multiplexing according to primer and barcode sequences a total of approximately 469 mill remaining reads were 3′ and 5′ trimmed according to base quality. Sequences below a quality of 96% were removed. The number of sequences used for taxonomical classification was 339,826,026 reads. Out of these 99.9% of the sequences were classified according to the Greengene SSU database.

### I. Characterization of the Intestinal Microbiota in Göttingen Minipigs

**Figure 1:**
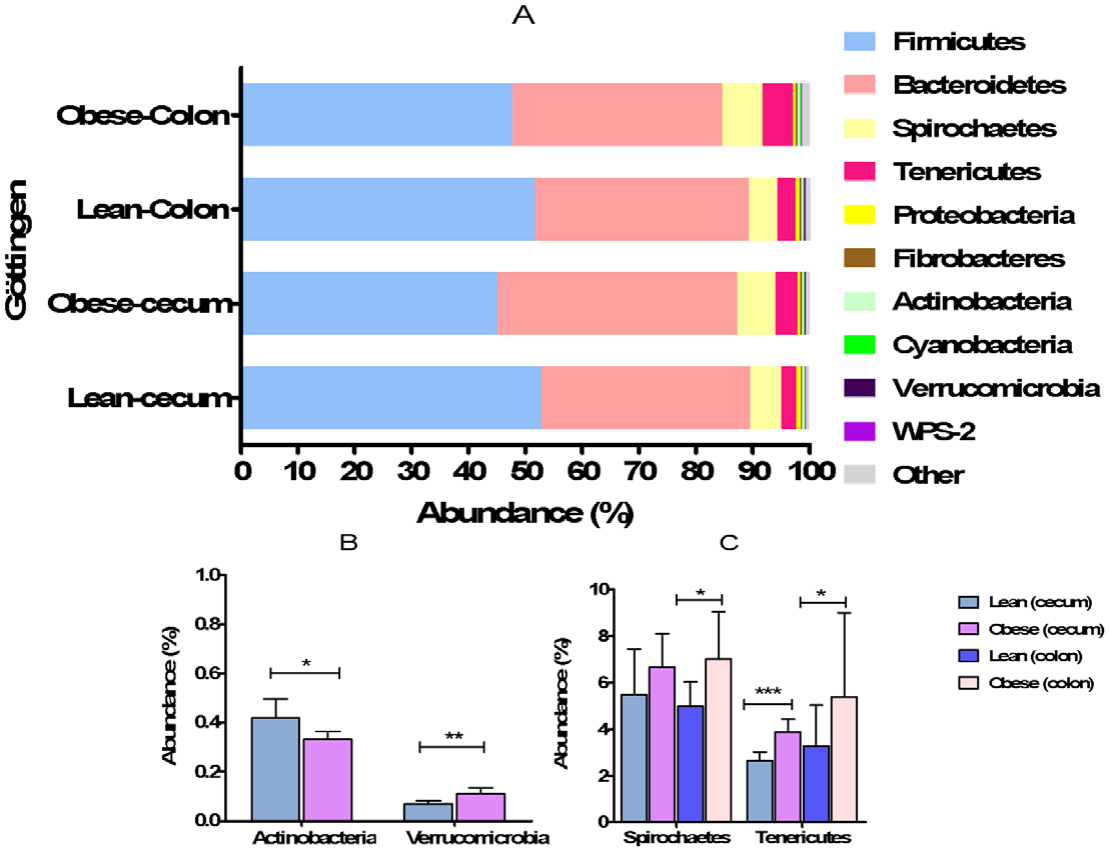
Abundance of phyla in colonic and cecal microbiota of lean and obese Göttingen minipigs (A–C). Different phyla in colon and cecum of lean and obese Göttingen minipigs. The error bars represent standard deviations.

In Göttingen minipigs the five most abundant phyla constituted 98.1% of all the obtained phyla (Figure 1A). The most abundant sequences obtained at phyla level were the Firmicutes consisting of 49.3% of all the normalized reads and the second most abundant phyla, the Bacteroidetes constituted 38.3% of all the normalized reads. Spirochaetes, Tenericutes and Proteobacteria constituted 6%, 3.8% and 0.6% of all the normalized reads, respectively. The diversity analysis of colonic and cecal microbiota from the obtained sequences at genus level showed no difference between lean and obese Göttingen minipigs.

At phyla level, the abundance of Firmicutes was higher in cecal microbiota of lean minipigs than the obese minipigs (*P<*0.006) while no significant difference was observed in Bacteroidetes between the two groups of minipigs (Figure 1A).

A higher abundance of Actinobacteria was observed in lean cecal microbiota of Göttingen minipigs (*P<*0.01) compared to obese minipigs (Figure 1B). In other phyla, there was a significant difference in the abundance of *Verricomicrobia* between lean and obese minipigs’ cecum (*P<*0.005), with the obese cecum containing a higher abundance of the bacteria belonging to this phylum (Figure 1B). The abundance of Spirochaetes was higher in colonic microbiota of obese minipigs (*P<*0.03) as compared to lean minipigs (Figure 1C). The abundance of Tenericutes was higher in both cecal and colonic microbiota of obese minipigs as compared to lean minipigs (*P<*0.004, *P<*0.04, respectively) (Figure 1C). In both lean and obese minipigs, the cecal microbiota consisted mainly of Firmicutes while the colonic microbiota had a higher abundance of Bacteroidetes. The ratio of Firmicutes to Bacteroidetes was significantly higher in cecal (*P<*0.0005) and colon (*P<*0.0001) microbiota of lean Göttingen minipigs (1.5 and 1.3, respectively).In obese Göttingen minipigs only cecal microbiota had higher ratio of Firmicutes to Bacteroidetes (1.3) (*P<*0.0006).

**Figure 2:**
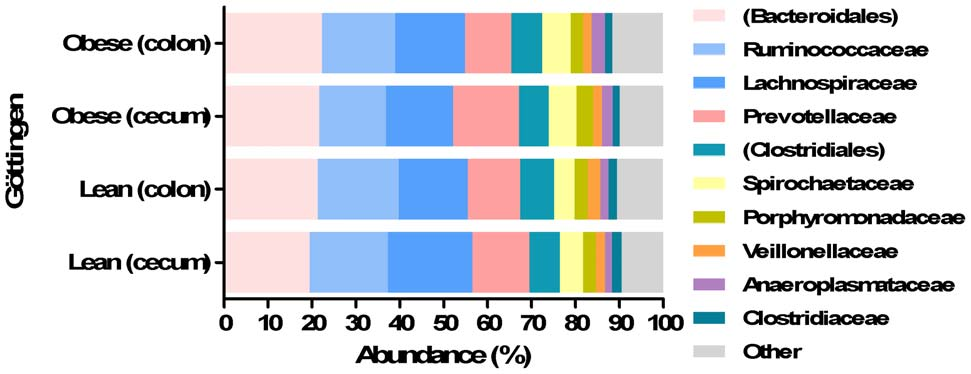
Abundance of different bacterial families in colonic and cecal microbiota of lean and obese Göttingen minipigs. Different families in colon and cecum of lean and obese Göttingen minipigs. Parentheses indicate unknown family.

At family level, the 15 most abundant families of bacteria constituted 94.7% of all bacteria and unknown *Bacteroidales*, *Ruminococcaceae*, *Lachnospiraceae*, *Prevotellaceae* and unknown family belonging to order *Clostridiales* were the most abundant families (Figure 2). There was no difference in the microbiota in neither colon nor cecum between lean and obese minipigs in abundance of *Lactobacillaceae* and *Enterobacteriaceae*. A higher abundance of *Streptococcaceae* (*P<*0.02) and *Bifidobacteriaceae* (*P<*0.04) was observed in cecum samples from lean minipigs compared to obese minipigs. The abundance of *Verrucomicrobiaceae* was higher in colonic microbiota of obese minipigs than the lean minipigs (*P<*0.01). There were several differences between lean and obese cecal microbiota in bacteria belonging to different families (Figure 2).

**Figure 3:**
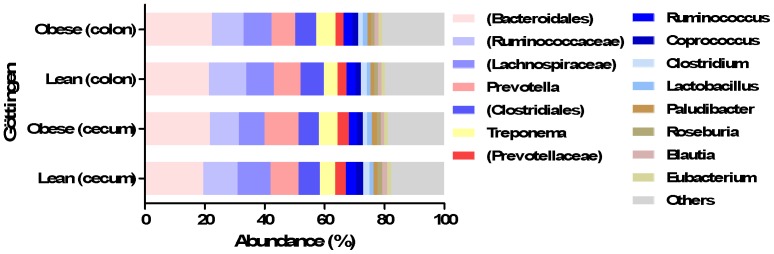
Abundance of different genera in colonic and cecal microbiota of lean and obese Göttingen minipigs. Different genera in colon and cecum of lean and obese Göttingen minipigs. Parentheses indicates unknown genus belonging to an order or a family.

At genus level, the 50 most predominant genera consisted of 97.5% of the entire genera represented from all the normalized reads. The most predominant genera were unknown genera belonging to order *Bacteroidales*, families *Ruminococcaceae* and *Lachnospiraceae* (Figure 3). The genus *Prevotella* constituted 9.4% of all the normalized reads. Several genera belonging the phylum Tenericutes were significantly higher in the obese cecal microbiota such as genera belonging to the families *Anaeroplasmataceae* (*P<*0.001), *RF39* (*P<*0.01) and *Erysipelotrichaceae* (*P<*0.001). Of other bacteria in obese cecal microbiota with significant difference were *Sphaerochaeta* (*P<*0.05), unknown genus belonging to family *alpha-Proteobacteria* (*P<*0.001), *YS2* (*P<*0.001), unknown genus belonging to order *Pirellulales* (*P<*0.001) and unknown genus belonging to phylum Tenericutes (*P<*0.001). *Methanobrevibacter* (*P<*0.01) and unknown genus belonging to *beta-Proteobacteria* in family *Alcaligenaceae* had a higher abundance in lean cecal microbiota. The lean minipigs had a higher abundance of *Clostridium* in cecum as compared to the obese minipigs (*P<*0.01) and the cecal microbiota of obese minipigs had a higher abundance of *Bacteroides* compared to lean minipigs (*P<*0.01). A higher abundance of the genus *Akkermensia* was observed in cecum of lean minipigs as compared to obese minipigs (*P<*0.01). The colonic and cecal microbiota of obese minipigs had a different abundance of *Prevotella* which were higher in obese cecal microbiota (*P<*0.0001).

### II. Characterization of the Intestinal Microbiota in Ossabaw Minipigs

**Figure 4:**
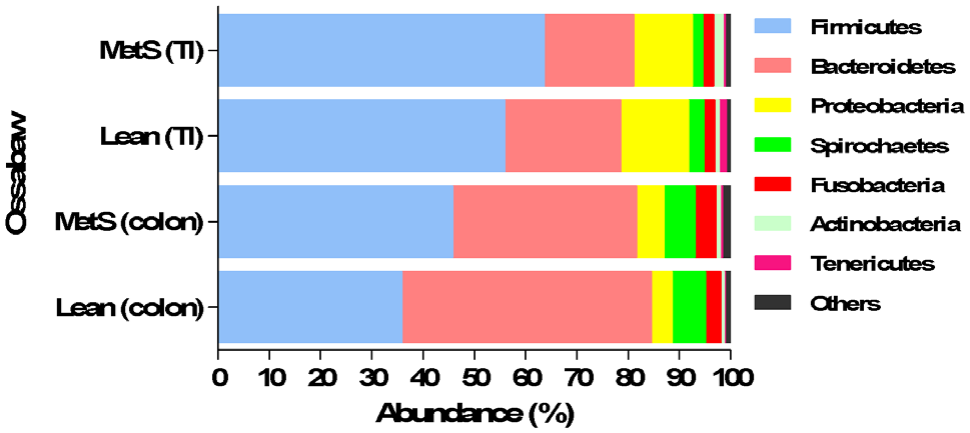
Microbial profiles of colon vary between lean and obese (MetS) Ossabaw minipigs. Selected phyla in terminal ileum (TI) and colon of lean and obese Ossabaw minipigs.

To characterise the intestinal microbiota of lean and obese Ossabaw minipigs, the colonic and ileal microbiota were sequenced. Overall, the ten most abundant phyla constituted 99.7% of all the phyla obtained from Illumina sequencing (Figure 4). The six most abundant phyla, constituted 98.4% of all the phyla obtained. These phyla were the Firmicutes consisting of 54.6% of all the normalized reads followed by Bacteroidetes with 27.3% of all the normalized reads. The next most abundant phyla were Proteobacteria (8.4%), Spirochaetes (3.6%), Fusobacteria (3%), Actinobacteria (1.3%) and Tenericutes (0.5%), respectively. The diversity analysis of colonic and ileal microbiota from the obtained sequences at genus level showed no difference between lean and obese Ossabaw minipigs.

A higher relative abundance of Firmicutes and a relative lower abundance of Bacteroidetes were observed in both colon and ileal microbiota in obese minipigs as compared to lean minipigs. The abundance of Bacteroidetes and Firmicutes were reversed in lean Ossabaw minipigs, with colon microbiota of lean Ossabaw minipigs consisting of 48.7% Bacteroidetes and 35.8% Firmicutes, while the obese Ossabaw minipigs had 45.7% Firmicutes and 35.9% Bacteroidetes in their colonic microbiota. Within the lean Ossabaw minipigs, a higher relative abundance of Bacteroidetes was observed in colon compared to terminal ileum. The obese Ossabaw minipigs had a higher ratio of Firmicutes to Bacteroidetes in terminal ileum and colon (3.9 and 1.3, respectively) as compared to lean Ossabaw minipigs (2.5 and 0.7, respectively).

There was a trend towards higher abundances of Proteobacteria and Spirochaetes in obese minipigs’ colon as compared to lean.

At class level the 10 most abundant classes constituted 98.2% of all the classes obtained from the normalized reads. Statistical analysis was performed on *Clostridia*, *Bacteroidia*, *Bacilli*, *alpha*-, *beta*-, *delta*-, *gamma, epsilon*-*Proteobacteria*, *Mollicutes* and Actinobacteria. There was a higher abundance of *Clostridia* in colon of obese Ossabaw minipigs as compared to lean Ossabaw minipigs colonic microbiota and a higher abundance of *Bacteroidia* in lean colon with 48.5% (±9.8) of all bacteria as compared to colon samples from obese Ossabaw minipigs (35.5% (±14)). The class *Bacilli* (phylum Firmicutes*)*, were highest in abundance in terminal ileum of obese minipigs. Overall the abundance of *beta-Proteobacteria* was higher in the microbiota from terminal ileum of both lean and obese minipigs compared to colon.

**Figure 5:**
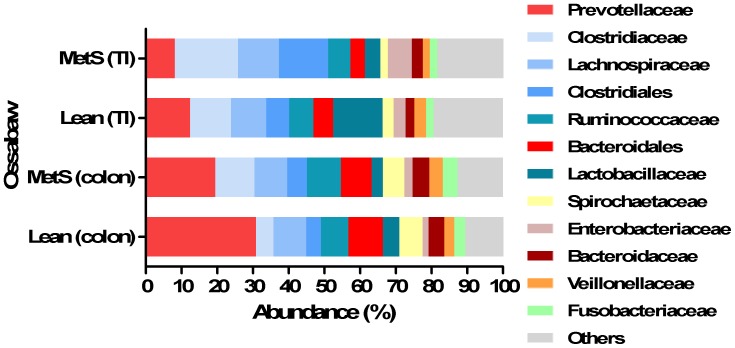
Different microbial profiles in microbiota of lean (Lean) and obese Ossabaw minipigs (MetS) at family level. Different phyla in terminal ileum (TI) and colon of lean and obese Ossabaw minipigs.

The families *Prevotellaceae*, *Clostridiaceae* and *Lachnospiraceae* together constituting 39.2% of all the normalized reads obtained at family level (Figure 5). *Prevotellaceae* was more abundant in lean Ossabaw minipigs than in obese Ossabaw minipigs with a mean relative abundance of 30.8% and 19.4%, respectively (Figure 5). There was a higher abundance of *Lactobacillaceae* in terminal ileum of lean minipigs compared to obese minipigs. The abundance of *Streptococcaceae* was highest in terminal ileum of lean minipigs and generally higher in terminal ileum than colon (Figure 5).

**Figure 6:**
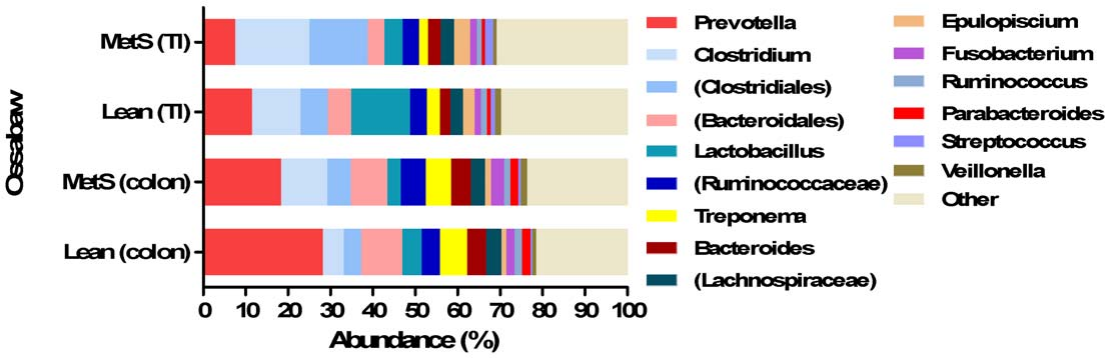
Abundance of different genera in colonic and ileal microbiota of lean and obese (MetS) Ossabaw minipigs. Selected genera in terminal ileum (TI) and colon of lean and obese Ossabaw minipigs. The unknown genera are indicated by parentheses belonging to the corresponding order or family.

At genus level, the 50 most predominant genera consisted of 93.1% of the entire genera present in all normalized reads. The two most abundant genera were *Prevotella* and *Clostridium* both consisting of 16.1% and 11.7% of all the normalized reads, respectively (Figure 6). In lean minipigs, the colonic microbiota had a higher abundance of *Prevotella* with a mean relative abundance of 28% (±5.5) while the obese minipigs had a mean relative abundance of 18.3% (±4.0) (Figure 6). However, *Clostridium* was more abundant in colonic microbiota of obese minipigs than lean minipigs (Figure 6). *Lactobacillus*, the fifth most abundant genus obtained from all the normalized reads, constituted 5% of the entire genera. There was a difference between the abundance of *Lactobacillus* within the microbiota of colon of lean Ossabaw minipigs having a higher abundance than the microbiota of obese Ossabaw minipigs (Figure 6). The abundance of *Streptococcus* in colon and terminal ileum was higher in obese Ossabaw minipigs than in lean Ossabaw minipigs (Figure 6). Overall, the abundance of *Streptococcus* was higher in terminal ileum than colon in both lean and obese group. There were several differences in bacteria belonging to different genera between the microbiota in colon and terminal ileum in both lean and obese minipigs. Generally *Parabacteroides* was more abundant in colon than terminal ileum (Figure 6).

### III. Fold-differences between Lean and Obese Minipigs in Selected Groups of Bacteria

The obtained results from high throughput qPCR showed several differences between lean and obese minipigs in both Göttingen and Ossabaw minipigs. There were many groups of bacteria that were not detectable by this qPCR system, such as Verrucomicrobia, *Bifidobacteriaceae*, *Cl. cluster IV*, *delta*-, *beta*-, *gamma-Proteobacteria*, *Enterococcus* and *Escherichia coli*.

In the obese Göttingen colon microbiota, there was a 7.8-fold higher abundance of Bacteroidetes than in the lean minipigs (Table 2). The only significant difference was found in relative abundance of bacteria belonging to the *Cl. cluster XIV* family, with a 7.6-fold higher abundance in colon of obese Göttingen minipigs as compared to the lean group (*P<*0.0001).

**Table 2:** Fold-differences in the relative abundance of bacteria between lean and obese minipigs.

	Göttingen (cecum)	Göttingen (colon)	Ossabaw (TI)	Ossabaw (colon)
**Firmicutes**	1	N.D.	N.D.	N.D.
**Bacteroidetes**	<2	**7.6** *	<2	1
**Actinobacteria**	<1	<1	**5.0**	**5.5**
**Fusobacteria**	N.D.	1	N.D.	N.D.
**Spirochaetes**	**3.3**	N.D.	N.D.	N.D.
***epsilon-Proteobacteria***	N.D.	2	N.D.	N.D.
***Lactobacillaceae***	<1	<1	1	<1
***Streptoccocceae***	<1	<1	N.D.	N.D.
***Cl. Cluster IV***	<1	N.D.	<2	<1
***Cl.Cluster XIV***	<1	**7.6** *	<1	<1
***Bacteroides***	N.D.	N.D.	**4.6**	**4.3**
***Cl. Perfringens***	N.D.	**3.5**	N.D.	N.D.

Relative abundance of bacteria in different phylogenetic groups in cecal and colonic microbiota of obese Göttingen minipigs and in ileal and colonic microbiota of obese Ossabaw minipigs, estimated by qPCR dynamic array.

N.D. stands for Non Detectable.

* indicates values with significant difference of *P<*0.05.

Actinobacteria had a 5-fold higher relative abundance in colon and terminal ileum of obese Ossabaw minipigs than lean minipigs. A 4-fold higher abundance of bacteria belonging to the genus *Bacteroides* was observed in terminal ileum and colon of obese Ossabaw minipigs (Table 2).

## Discussion

In this study we aimed to characterise the composition of the intestinal microbiota of two minipig models of obesity and the effect of diet and adiposity on gut microbial community in these minipigs. Göttingen minipigs have been used as obesity models and displayed minor abnormalities in glucose tolerance and insulin sensitivity [26], however the metabolic changes in lean and obese Göttingen minipigs on the diets used in the present study are not well described. Ossabaw minipigs have been used as animal models in studies of obesity related metabolic disorders such as cardiovascular disease and are predisposed to insulin resistance in response to obesity and high-fat feeding, which is unique in Ossabaw minipigs as opposed to other animal models [22]. Lee *et al*. [23] reported several differences in blood parameters such as cholesterol and triglycerides between lean and obese Ossabaw minipigs. The Ossabaw minipigs used in our study displayed metabolic syndrome with abdominal and visceral adiposity, high blood triglyceride levels and high total blood cholesterol [23]. To our knowledge the Ossabaw and Göttingen minipigs’ intestinal microbiota has not been characterised before and this is the first study that investigated the relation between obesity and intestinal microbial community in Ossabaw and Göttingen minipigs by 16S rRNA gene sequencing using the Illumina technology.

In this study we characterise the Göttingen minipigs’ cecal and colonic microbiota in relation to obesity which was induced by overeating and not high-fat/high-energy diet. In the cecum microbiota of Göttingen minipigs there were several differences related to their metabolic state, while no differences were seen in the microbiota in colon. Interestingly the ratio of Firmicutes to Bacteroidetes was opposite to the findings in Ossabaw minipigs, as the lean Göttingen minipigs had a higher abundance of Firmicutes relative to Bacteroidetes. These results are however in agreement with the findings in diabetic mice and humans [7], [36]. One of the interesting findings was the higher abundance of bacteria belonging to phylum Tenericutes (class *Mollicutes*) in obese Göttingen minipigs compared to their lean counterparts. The bacteria belonging to this phylum have previously been shown to be increased in diet-induced obese mice [9]. Noteworthy was the differences observed in *Bacteroides* between lean and obese Göttingen minipigs, with higher abundances in obese minipigs which has previously been reported to be connected to gut microbiota of T2D in humans [7]. In brief, the cecal microbiota of obese Göttingen minipigs was different from that of lean minipigs with higher abundance of bacteria belonging to phyla Bacteroidetes, Spirochaetes, Tenericutes and Verrucomicrobia, indicating a specific gut microbial profile in obese Göttingen minipigs. We also identified genera with higher abundances in lean Göttingen minipigs, namely *Akkermensia* and *Methanovibrobact*er. Species belonging to *Akkermensia* have been shown to play a protective role in the gut microbiota of mice against autoimmune disease such as type 1 diabetes [37] and species belonging to the *Methanovibribacter*, have been connected to a lean phenotype [38]. Recent studies have shown that the species *Methanovibribacter smithii* co-occurs with several other species such as species in the genus *Clostridia* [39].

In Ossabaw minipigs, we report that the gut microbiota in relation to obesity was different between lean and obese Ossabaw minipigs. Our data suggest that obesity and/or HFD affected the gut microbiota of obese Ossabaw minipigs, since they had an overall different gut microbial community as compared to their lean counterparts. HFD has previously been shown to affect the gut microbiota both in obesity and independently of the obese state [11] and ingestion of HFD has been implicated in causing metabolic disorders observed in obese pigs and mice [26], [40]. In our study the obese Ossabaw minipigs received HFD which could also have caused the differences observed between lean and obese Ossabaw minipigs. At phylum level the only differences between the lean and obese Ossabaw minipigs were observed in abundance of Firmicutes and Bacteroidetes, with higher abundance of Firmicutes in obese minipigs and higher abundance of Bacteroidetes in lean minipigs. Our results are in agreement with studies in obese humans [8], mice [6] and pigs [19] that displayed only obesity, while the results contradicts several human studies in subjects with T2D [7], [41]. However, at lower taxonomic levels, more differences were observed between lean and obese Ossabaw minipigs. A marked difference was observed in the abundance of the families *Prevotellaceae* and *Lactobacillaceae*, especially genera *Prevotella* and *Lactobacillus*, which had higher abundances in lean Ossabaw minipigs while these results are opposite to the findings reported previously with higher abundance of *Prevotella* in diabetic humans and mice [7], [36]. Our results are however, in agreement with those reported by Turnbaugh *et al*. indicating that the state of obesity shapes the gut microbiota [10]. Nonetheless, diet is also a factor that may have caused the changes we observed here. Furthermore, the abundance of *Lactobacillus* was significantly lower in colon of obese Ossabaw minipigs as compared to lean Ossabaws and this result has been shown before in mice fed a HFD [42], implicating diet as a factor that shapes the intestinal microbiota.

HFD has been implicated in changing the gut permeability by modulating the gut microbiota. A positive correlation has been observed between higher abundances of *Lactobacillus* and colonic transepithelial resistance [42]. The lean Ossabaw minipigs had a higher abundance of *Lactobacillus* in their colonic microbiota compared to obese and did not display metabolic dysfunction as compared to obese Ossabaw minipigs. The low abundance of *Lactobacillus* observed in obese Ossabaw minipigs could have caused changes in their intestinal permeability; however the intestinal permeability was not measured in our study. Increased gut permeability has been postulated to be related to metabolic endotoxemia, causing metabolic dysfunctions such as T2D. In case of *Lactobacillus*, the gut microbiota may have contributed in shaping the metabolic profile observed in obese Ossabaw minipigs by changing the gut permeability. Together our results suggest that the obese Ossabaw minipigs’ gut microbiota display a profile that resembles the intestinal microbiota observed in obese mice and humans in response to HFD.

According to our data, the gut microbiota of obese Ossabaw minipigs displays the characteristic of obese gut microbiota and not MetS. One explanation for these results could be that these minipigs had to receive the HFD for a longer time in order to induce insulin resistance in them. However, it must be mentioned that the data from Ossabaw minipigs are based on a small number of animals and therefore, a larger population would provide a better understanding of the Ossabaw minipigs gut microbial community.

Obese Göttingen minipigs display a specific gut microbiota that has previously been connected to metabolic disorders such as T2D. Even though obese Göttingen minipigs do not display metabolic disorders to the extent of that of obese Ossabaw minipigs, their gut microbial profile have similarities to those found previously in T2D mice. Therefore, Göttingen minipigs have great potential for being used in obesity related gut microbial studies. Here it is reported that certain group of bacteria flourish best under HFD conditions such as Firmicutes as observed in Ossabaw minipigs, while others such as the genus *Bacteroides* flourished due to obesity under other diet conditions such as overeating as observed in obese Göttingen minipigs.

Together, these findings can be used to approach the obesity-related gut microbiota by exploring how the microbial community co-occur in periods of high energy feed and how this diet affects the community and its’ stability over a period of time. Such information can be used to develop probiotic strains to change the gut microbial community into a composition that protects against increased gut permeability, low grade inflammation, obesity and the obesity-related metabolic disorders.

In conclusion, obesity is connected to radical changes in several bacterial groups as observed in obese Göttingen minipigs. Our data suggest diet as an important factor that shapes the gut microbial community as shown in Ossabaw minipigs. However, it must be kept mind that our data is based on low number of animals and the Ossabaw study needs further investigation in a larger population. Here we show that both Ossabaw and Göttingen minipigs may be useful animal models in studying gut microbiota and provide a better understanding of obesity and metabolic syndrome and the relationship to the gut microbiota. Together, these findings can be used to approach the obesity-related gut microbiota to develop probiotic strains.

## Supporting Information

Figure S1 **Weight progression in obese Göttingen minipigs over a period of 122 days.**
(TIF)

Figure S2 **Weight progression in obese Ossabaw minipigs in duration of 32 weeks.**
(TIF)
